# Wnt5a is a crucial regulator of neurogenesis during cerebellum development

**DOI:** 10.1038/srep42523

**Published:** 2017-02-16

**Authors:** Chandramohan Subashini, Sivadasan Bindu Dhanesh, Chih-Ming Chen, Paul Ann Riya, Vadakkath Meera, Thulasi Sheela Divya, Rejji Kuruvilla, Kerstin Buttler, Jackson James

**Affiliations:** 1 Neuro Stem Cell Biology Laboratory, Neurobiology Division, Rajiv Gandhi Centre for Biotechnology, Thiruvananthapuram, Kerala-695 014, India; 2 Department of Biology, Johns Hopkins University, 3400 N. Charles St., 224 Mudd Hall, Baltimore, MD 21218, USA; 3 Department of Anatomy and Cell Biology, University Medicine Göttingen, 37075-Göttingen, Germany

## Abstract

The role of Wnt5a has been extensively explored in various aspects of development but its role in cerebellar development remains elusive. Here, for the first time we unravel the expression pattern and functional significance of Wnt5a in cerebellar development using Wnt5a^−/−^ and Nestin-*Cre* mediated conditional knockout mouse models. We demonstrate that loss of Wnt5a results in cerebellar hypoplasia and depletion of GABAergic and glutamatergic neurons. Besides, Purkinje cells of the mutants displayed stunted, poorly branched dendritic arbors. Furthermore, we show that the overall reduction is due to decreased radial glial and granule neuron progenitor cell proliferation. At molecular level we provide evidence for non-canonical mode of action of Wnt5a and its regulation over genes associated with progenitor proliferation. Altogether our findings imply that Wnt5a signaling is a crucial regulator of cerebellar development and would aid in better understanding of cerebellar disease pathogenesis caused due to deregulation of Wnt signaling.

Cerebellum is a rhombomere1 derivative that controls motor functions and higher cognitive functions^1^,^2^. It is known for its highly foliated and well-defined cytoarchitecture that makes it a suitable model system for understanding various mechanisms behind the genesis and maturation of different subtypes of neurons. Different neuronal subtypes are generated in a very sequential manner both during the embryonic and postnatal development from two distinct primary germinal centers, the ventricular zone (VZ)^3^ and the rhombic lip (RL)^1^. VZ is demarcated by the defined expression of specific transcription factors such as Ptf1α, Mash1, Neurogenins (Ngn)^4^,^5^, while RL is defined by the expression of Math1 and Pax6^6^,^7^. During postnatal development VZ delaminates to give rise to secondary germinal center, the prospective white matter (PWM)^8^,^9^, and the RL progenitors that migrate tangentially above the subpial surface giving rise to external granular layer (EGL)^10^. Moreover, the VZ progenitors also gives rise to all GABAergic neurons and glial cells of the cerebellum while RL progenitors gives rise to all the glutamatergic neuronal subtypes^4^,^7^,^10^. The correct type, location and number of neurons are generated by the interplay of various signaling molecules and transcription factors ensuring proper cerebellar development. One of the key signaling pathways that are known to exert crucial role in regulating various aspects of neurogenesis is Wnt signaling^11^. Wnt signaling proteins are lipid modified glycoproteins that are highly conserved among various species. To date almost 19 Wnt ligands are known that mediate important functions during development^12^,^13^,^14^. Based on the ability to activate β-catenin, Wnt signaling can be classified into canonical and non-canonical pathway^15^. Majority of the Wnt ligands mediate canonical pathway i.e β-catenin dependent pathway while some ligands such as Wnt4, Wnt5a and Wnt11 mediate non-canonical Wnt signaling i.e β-catenin independent pathways^16^,^17^,^18^. In cerebellum, Wnt/β-catenin signaling has been shown to promote the proliferation of VZ progenitors and impair their differentiation during early development^19^. Other studies have identified the role of Wnt7a and Wnt3 in regulating axon genesis and differentiation of CGN progenitors respectively^20^,^21^. Additional support for role of Wnt β-catenin signaling in cerebellar development comes from its association with cerebellar associated tumors, medulloblastoma. Though several studies have clearly demonstrated the function of canonical Wnt signaling in development and disease pathogenesis, role of non-canonical Wnt signaling in cerebellar neurogenesis is just beginning to be uncovered. Recently, role for non-canonical Wnt signaling has been suggested in medulloblastoma pathogenesis. Further Wnt5a, a classic non-canonical Wnt ligand has been shown to be expressed highly in medulloblastoma tumor samples but its role in cerebellar development remains obscure. Wnt5a being one of the well characterized non-canonical Wnt ligand with key roles during cortical and midbrain neurogenesis^22^,^23^,^24^, it is prudent to look at the role of Wnt5a signaling in cerebellar development.

Here, we show that Wnt5a is robustly expressed in mouse cerebellum during prenatal and postnatal developmental stages. Additionally, we show that loss of Wnt5a leads to significant reduction in VZ derived GABAergic neurons and RL derived early born glutamatergic subtypes such as glutamatergic neurons of deep cerebellar nuclei (DCN) and unipolar brush cells (UBC’s) due to reduction in radial glial and granule neuron progenitor cell proliferation thereby resulting in cerebellar hypoplasia. Thus, our study for the first time demonstrates the functional role of Wnt5a in mediating cerebellar development and suggests that Wnt5a signaling is an essential regulator of growth and development of cerebellum.

## Results

### Wnt5a is robustly expressed in cerebellum during prenatal and postnatal development

**Figure 1:**
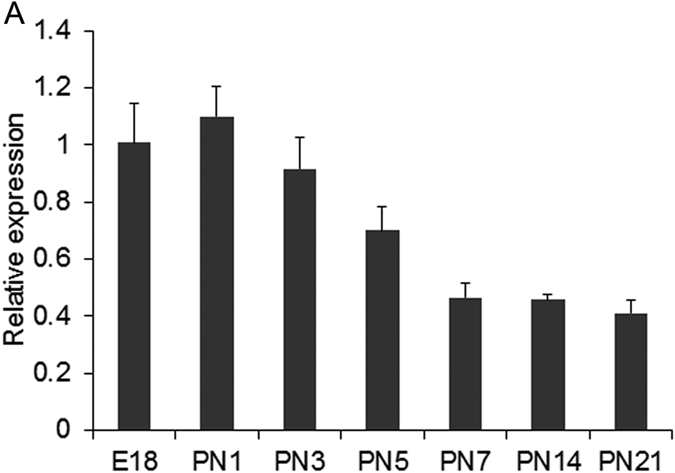
Temporal expression of Wnt5a in prenatal and postnatal mouse cerebellum. (**A**) Real-Time PCR analysis of Wnt5a expression in mouse cerebellum from E18 to PN21 stages showed an increase in Wnt5a expression from E18-PN3 stages which gradually decreases as development proceeds. Data expressed as Mean ± SD, *n* = *3.*

Though the expression and pleiotropic function of Wnt5a is well evidenced *in vitro*^23^,^25^ and *in vivo*^22^,^26^ specifically in cortical and midbrain neurogenesis, its expression pattern and functional implications during cerebellar development still remains ambiguous. Therefore, to understand the temporal expression of Wnt5a during cerebellar development, we carried out real time PCR analysis starting from E18 to PN21. Our results showed that Wnt5a was expressed throughout the development with peak expression between E18 to PN1 (Fig. 1A) as compared to late postnatal stages (PN5-PN21), consistent with previous report showing that Wnt5a expression decreases during postnatal development^20^. Thus these observations clearly confirmed the expression of Wnt5a in cerebellum during the embryonic and postnatal development strongly suggesting a possible role for Wnt5a in regulating cerebellar neurogenesis.

### Loss of Wnt5a leads to cerebellar hypoplasia and lobulation defects

**Figure 2:**
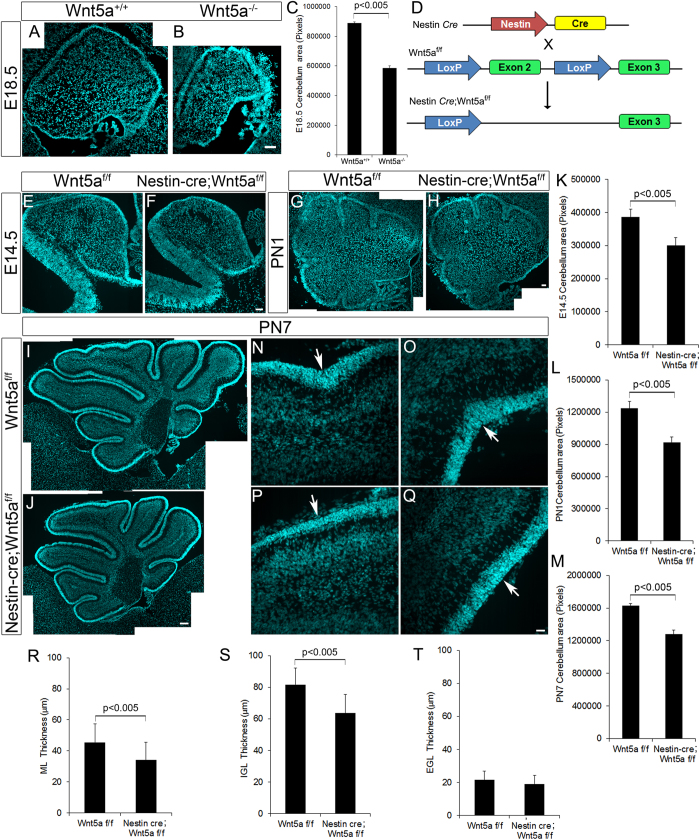
Conditional ablation of Wnt5a results in cerebellar hypoplasia and lobulation defects. (**A**,**B**) DAPI staining in sagittal sections of Wnt5a^+/+^ (**A**) and Wnt5a^−/−^ (**B**) cerebellum at E18.5 stage. (**C**) Quantitative measurement of cross sectional area of cerebellum at E18.5. (**D**) Schematic showing the generation of Nestin-*Cre* conditional *Wnt5a* mutant mice. (**E**–**J**) DAPI staining in sagittal sections of Wt-type (Wnt5a^f/f^) and Wnt5a cKO (Nestin-*cre*; Wnt5a^f/f^) cerebellum at E14.5 (**E**,**F**), PN1 (**G**,**H**) and PN7 (**I**,**J**) stages. (**K**–**M**) Quantitative measurement of cross sectional area of cerebellum at E14.5 (**K**), PN1 (**L**) and PN7 (**M**). (**N**–**Q**) DAPI staining showing lobulation defects in PN7 *Wnt5a* cKO cerebellum, arrows point to the absence in fissure separating lobule VIa and VIb, IXa and IXb in Wnt5a cKOs compared to control. (**R**–**T**) Quantification of different cortical layer thickness in PN7 cerebellum of wild type and Wnt5a cKO mice. Image (**G**–**J**) is generated by stitching together multiple images using Photoshop software. Data expressed as Mean ± SD, *n* = *3*. Scale bar = 50 μm (**A**,**B**), 50 μm (**E**–**J**), 25 μm (**N**–**Q**).

In order to understand the functional relevance of Wnt5a during cerebellar development we used Wnt5a^−/−^ null mutant transgenic mice model. At E18.5 Wnt5a^−/−^ null mutants displayed significant reduction in cross-sectional area of the cerebellum as compared to the wild-type control (p < 0.005, Fig. 2A,B & C). Additionally, the four principal fissures were evident in the control (Fig. 2A) while in the null mutant cerebellum these fissures were not observed and the cerebellum remained non-lobulated (Fig. 2B). Thus, these results indicated the essential role of Wnt5a in ensuring normal cerebellar development.

Since Wnt5a^−/−^ null mutants die prenatally, further analysis during the postnatal stages when cerebellar neurogenesis is active was precluded. Therefore, in order to circumvent this issue, we used Nestin-*cre* mediated Wnt5a conditional knockout mice model (Wnt5a cKO, Fig. 2D) for further studies, where Wnt5a expression is ablated in all the nestin expressing neural progenitors right from E10.5. Loss of Wnt5a expression in conditional knockout cerebellum as compared to the wild-type controls was confirmed by *In situ* hybridization using Wnt5a specific probes (Fig. S1). In line with the observations from Wnt5a^−/−^ null mutants, histological analysis of midsagittal sections of E14.5 Wnt5a cKO cerebellum showed significant reduction in the cross-sectional area of the cerebellum as compared to the wild-type siblings (Fig. 2E,F & K, p < 0.005). Similarly, at early postnatal PN1 and PN7 stages, we still continued to observe marked significant reduction in area of cerebellum in mutants as compared to wild-type siblings (Fig. 2G–J & L–M, p < 0.005 respectively). However, all the cortical layers remained intact and no observable defects in the lamination pattern was observed in conditional mutants with intact organization of glial fibers (data not shown). Even though all the cortical layers were clearly distinguishable, as there was notable reduction in size we measured the thickness of each cortical layer at PN7. We observed a significant reduction (p < 0.005) in the thickness of molecular layer (ML) and IGL in anterior and posterior lobes of cerebellum in mutants as compared to the wild-type littermates but we did not detect any significant difference in the EGL thickness (Fig. 2R–T). In addition, defects in lobulation pattern accompanied by reduction in depth of all the fissures in the mutants at PN7 were observed. Though the reduction in size was throughout the anterior posterior axis, lobulation defect was prominent in the central and posterior lobes. Declival sulcus, a shallow fissure that separates lobule VIa and VIb, and ulva sulcus, fissure separating IXa and IXb were missing in Wnt5a cKO compared to control (Fig. 2N–Q). These observations indicated the essential role of Wnt5a in mediating cerebellar development.

### Wnt5a regulates the generation of GABAergic neurons during the early embryonic and postnatal stages

**Figure 3:**
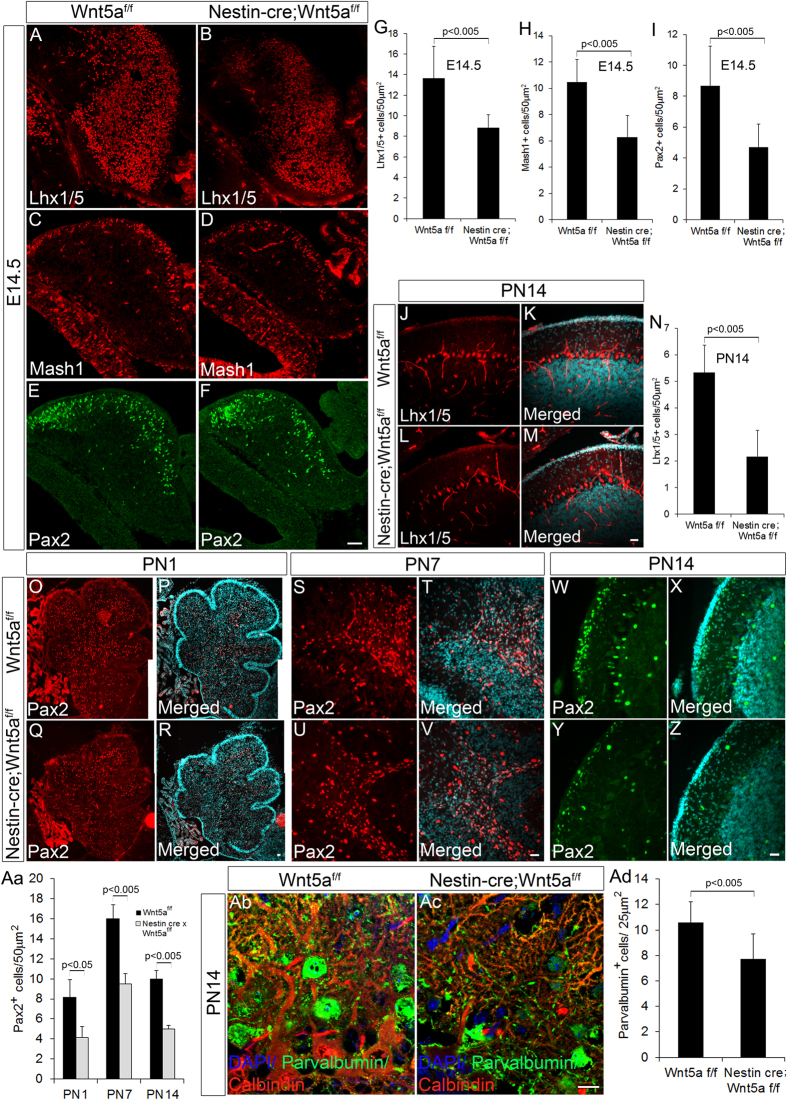
GABAergic neurogenesis is impaired in Wnt5a cKOs. (**A**–**F**) Immunohistochemical analysis of GABAergic neuronal markers Lhx1/5 (**A**,**B**), Mash1 (**C**,**D**) and Pax2 (**E**,**F**) at E14.5 stage in Wnt5a^f/f^ and Nestin-*cre*;Wnt5a^f/f^ embryos. (**G**–**I**) Quantification of Lhx1/5^+ve^ cells (**G**), Mash1^+ve^ cells (**H**) and Pax2^+ve^ cells (**I**) at E14.5. (**J**–**M**) Immunohistochemical analysis with Purkinje cell marker Lhx1/5 at PN14 stage. (**N**) Quantification of Lhx1/5^+ve^ cells at PN14. (**O–Z**) Immunohistochemical analysis of Pax2^+ve^ cells at PN1 (**O**–**R**) PN7 (**S**–**V**) and PN14 stages (**W**–**Z**). (**Aa**) Quantification of Pax2^+ve^ cells at PN1, PN7 and PN14. (**Ab**–**Ac**) Immunohistochemical analysis of Parvalbumin^+ve^ cells in the molecular layer at PN14. (**Ad**) Graph shows the quantification of parvalbumin^+ve^ molecular layer interneurons. Data expressed as Mean ± SD, *n* = *3.* Scale bar = 50 μm (**A**–**F**), 25 μm (**O**–**Z** & **Ab**–**Ac**)

GABAergic and glutamatergic neurons are the two major neuronal subtypes that occupy distinct layers of the cerebellum thereby conferring a well-defined and organized cyto-architecture to the cerebellum. Owing to the observed significant reduction in size of cerebellum and thickness of cortical layers, we assessed the expression of various factors that are known to mediate crucial roles in generation and differentiation of different GABAergic neuronal subtypes. Purkinje neurons (GABAergic projection neuron) and GABAergic interneurons of the DCN are born between E10.5 to E13.5. This is followed by GABAergic interneurons of IGL such as Golgi, born between E13.5-PN1 and finally basket and stellate ML interneurons respectively that are born between PN0-PN15^27^,^28^. Therefore, we first analyzed the expression of Lhx1/5, a LIM homeodomain factor that marks all the post-mitotic Purkinje neurons of the cerebellum^29^,^30^. Wnt5a cKOs displayed a significant reduction (p < 0.005) in the number of Lhx1/5^+ve^ Purkinje neurons at E14.5 (Fig. 3A,B & G) and PN14 (Fig. 3J–M & N) compared to controls. Further we examined if GABAergic interneuron that forms another major class of GABAergic neuron of the cerebellum was affected by loss of Wnt5a. Mash1 is one of the key factors that is known to play a pivotal role in generation of cerebellar GABAergic interneurons and is expressed by the VZ progenitors throughout development^31^,^32^. Interestingly, our results showed a prominent significant reduction (p < 0.005) in number of Mash1 expressing GABAergic progenitors in the Wnt5a cKO cerebellum at E14.5 stage (Fig. 3C,D & H). Subsequent investigation also revealed a significant reduction (p < 0.005) in number of Pax2^+ve^ GABAergic interneuron progenitors at E14.5 in Wnt5a cKOs as compared to the wild-type controls (Fig. 3E,F & I). Similarly, Wnt5a^−/−^ null mutants at E18.5 also exhibited severe reduction in Pax2^+ve^ GABAergic interneuron progenitors which further strongly supported the essential role of Wnt5a in GABAergic neurogenesis (Fig. S3D–F). This observation is strongly in agreement with our previous observation regarding the reduction in Mash1 expressing progenitors, and previous studies with Mash1 null mice that convincingly highlights the inevitable role of Mash1 in principally mediating the genesis of Pax2^+ve^ GABAergic interneurons of the cerebellum^32^. Since majority of Pax2^+ve^ GABAergic interneurons are generated during the first two postnatal weeks of mouse development we next evaluated the expression of Pax2 during these postnatal stages. Interestingly, we detected a significant reduction (p < 0.05) in Pax2^+ve^ GABAergic interneurons in Wnt5a cKOs at PN1 stage when almost all the Golgi interneurons occupying the IGL are born (Fig. 3O–R & Aa). Further analysis of Pax2^+ve^ cells at PN7 and PN14 stages, when the basket and stellate interneurons of the ML nears completion respectively, revealed a marked significant reduction (p < 0.005) in number of Pax2^+ve^ cells similar to that observed in PN1 stage (Fig. 3S–V & Aa,W–Z & Aa respectively). To further confirm these results, we used another marker parvalbumin that specifically marks the ML interneurons along with Purkinje cells present in cerebellum. The observation of a significant reduction (p < 0.05) in number of parvalbumin^+ve^ cells in the ML of Wnt5a cKOs as compared to the wild-type counterparts further validated our previous findings (Fig. 3Ab–Ac & Ad). Taken together, these observations clearly highlight the significance of Wnt5a signaling in regulating the genesis of GABAergic neurons in the cerebellum throughout development.

### Lack of Wnt5a expression reduces Purkinje cell dendritogenesis

**Figure 4:**
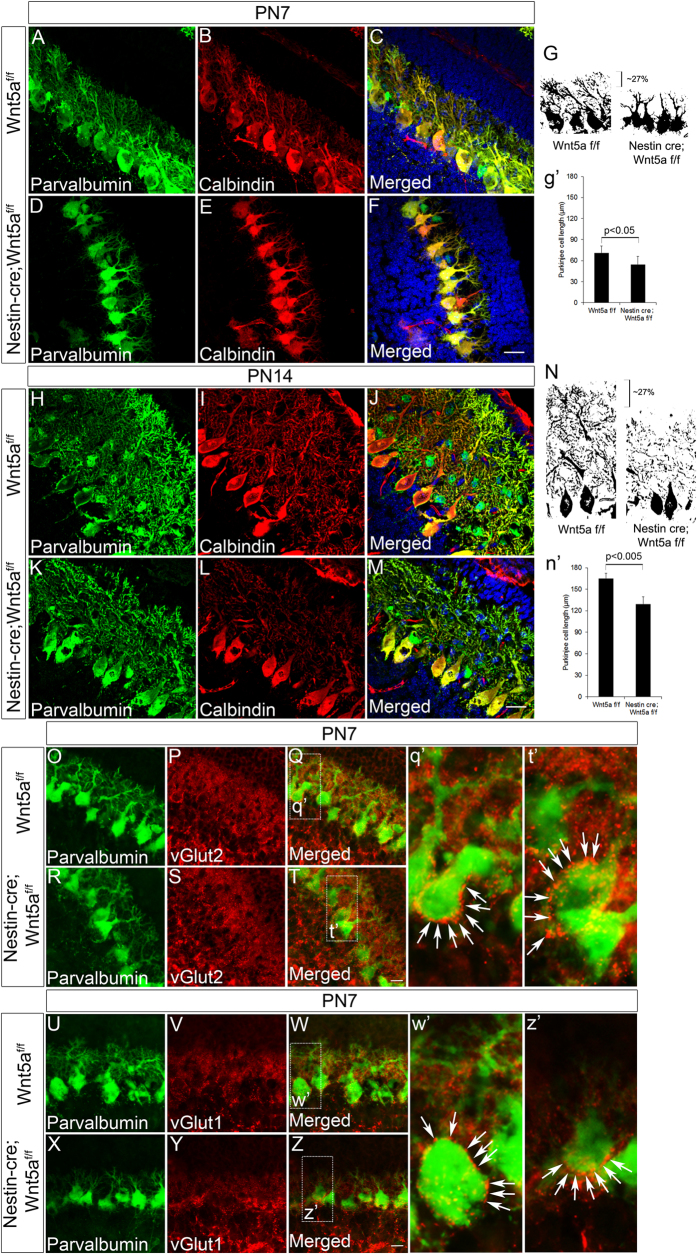
Purkinje neurons of the Wnt5a cKOs show stunted growth and exhibit dendritic branching defects. (**A**–**F**) Immunohistochemical staining with Purkinje cell specific marker calbindin and parvalbumin at PN7. (**H**–**M**) Immunohistochemical staining with Purkinje cell specific marker calbindin and parvalbumin at PN14 stage. (**G & g’**) Binary image of Purkinje cells of control and Wnt5a cKO immunostained with parvalbumin at PN7 stage, graph represents quantification of Purkinje cell length in μm. (**N & n’**) Binary image of Purkinje cells of control and Wnt5a cKO immunostained with parvalbumin at PN14 stage, graph represents quantification of Purkinje cell length in μm. (**O**–**T**) Co-immunostaining with VGlut2 and parvalbumin in midsagittal sections of Wt-type control and Wnt5a cKO cerebellum at PN7 stage. (**q’**,**t’**) Magnified view of selected area in Q and T and arrows indicate the VGlut2^+ve^ puncta around Purkinje Cell soma. (**U–Z**) Immunohistochemical analysis with VGlut1 and parvalbumin at PN7 stage. (**w’**,**z’**) Magnified image of selected area in W and Z and arrows indicate the VGlut1^+ve^ puncta on Purkinje Cell. Data expressed as Mean ± SD, *n* = *3.* Scale Bar = 25 μm

Since our previous results have demonstrated a notable reduction in ML thickness, a layer into which Purkinje neurons project and develop complex dendritic branches, we next focused on investigating the requirement of Wnt5a in regulating the differentiation of Purkinje neurons. Additionally, moderate expression during the postnatal stages also prompted us to check if Wnt5a had any role to play in Purkinje cell maturation since previous reports have described the importance of granule cells in regulating Purkinje cell development during postnatal stages^33^,^34^,^35^. Immunohistochemical analysis with calbindin and parvalbumin at PN7 stage indicated normal arrangement of Purkinje neurons in the Wnt5a cKOs as compared to the wild-type counterparts (Fig. 4A–F). This observation is supported by our previous findings where the number of Lhx1/5^+ve^ Purkinje neurons was reduced in the Wnt5a cKOs as compared to the wild-type siblings but without any defects in the migration pattern of Purkinje neurons. Even though, there was no difference in arrangement of Purkinje neurons, we did observe a marked significant reduction (p < 0.05) in dendritic branching of PN7 Wnt5a cKOs compared to wild-type counterparts (Fig. 4A–F & g’). Besides improper dendritic branching, these neurons remained stunted. Comparison of binary images showed that the reduction in dendritic length was ~27% in Wnt5a cKO cerebellum compared to Wt controls (Fig. 4G) indicating defects in Purkinje cell maturation. Further to check if the dendritic arborisation defects persisted during later stages of development, we analyzed the Purkinje cell morphology at PN14 using immunohistochemical analysis. Our results showed a consistent reduction in dendritic branching of Wnt5a cKO embryos compared to its wild-type counterpart (~27%, p < 0.005) which was almost similar to that observed at PN7 stage (Fig. 4H–M & N,n’). These observations clearly indicate that the alterations that are caused during early developmental stages due to lack of Wnt5a are faithfully reflected at later stages of development. Since the Purkinje cell differentiation is retarded in Wnt5a cKOs, we were also curious to know if an underdeveloped Purkinje neuron at PN7 would be capable of receiving innervations from other neurons. It is known that parallel fibers and climbing fibers innervate Purkinje neurons and establish synaptic connections with it^36^,^37^,^38^. Parallel fibers express vesicular glutamate transporter 1(VGlut1) whereas, climbing fibers express vesicular glutamate transporter 2 (VGlut2)^39^. Interestingly, immunohistochemical analysis with VGlut1 and parvalbumin indicated intact synaptic terminals in both Wt and Wnt5a cKOs showing no evident defects in innervations onto Purkinje cells (Fig. 4O–T & q’,t’). Similarly, immunostaining with VGlut2 and parvalbumin did not reveal any defects in climbing fiber innervations on to Purkinje cells in Wnt5a cKOs (Fig. 4U–Z & w’,z’). Therefore, from the above observations it appears that Wnt5a is a key factor involved in Purkinje cell maturation although its loss does not alter the synaptic connections onto Purkinje neurons.

### Conditional ablation of Wnt5a results in depletion of glutamatergic neurons of DCN and UBC

**Figure 5:**
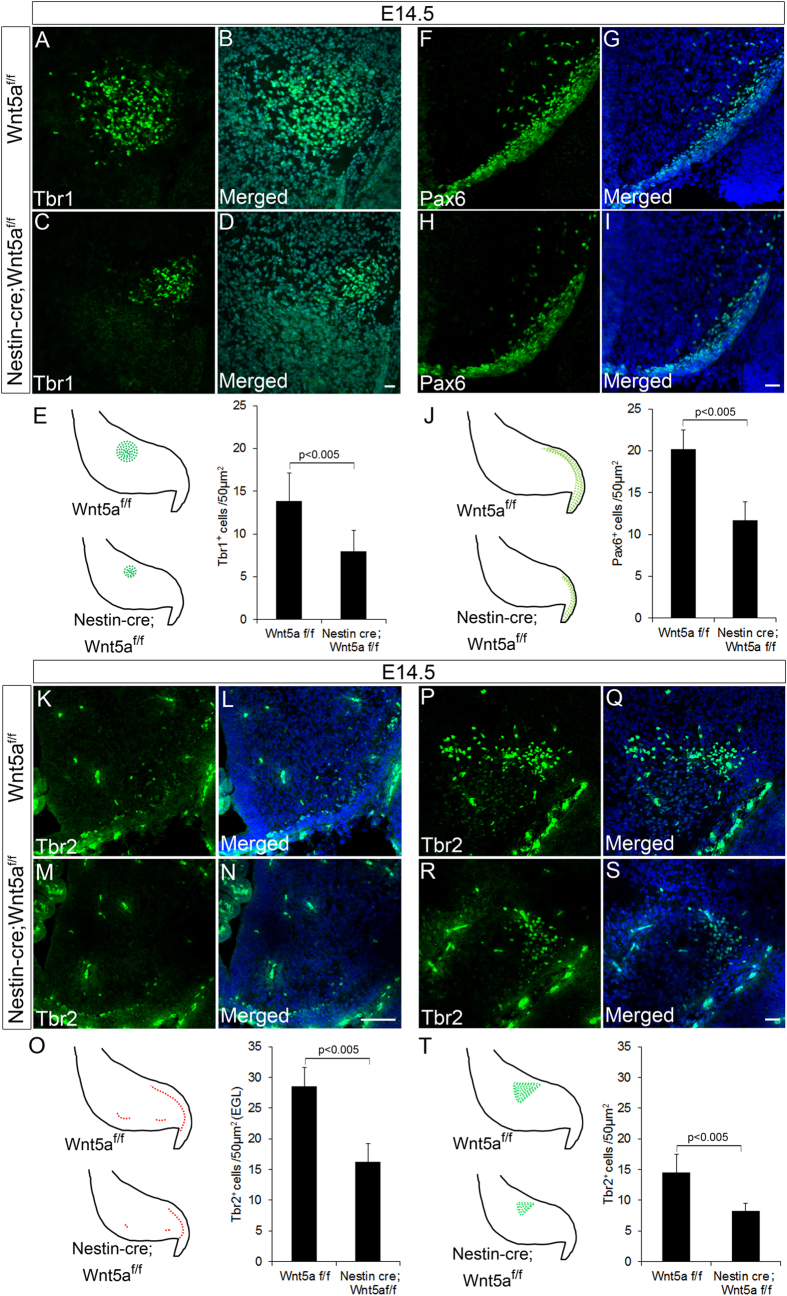
Rhombic Lip (RL) derived Glutamatergic neural progenitors are depleted at E14.5 in Wnt5a cKOs. (**A–I**) Immunohistochemical analysis of glutamatergic neuronal markers Tbr1 and Pax6 at E14.5. (**E**,**J**) Schematic showing the regions analyzed and represented in (**A**–**I**) and quantification of number of Tbr1^+ve^ cells in NTZ and Pax6^+ve^ granule neuron progenitors in EGL respectively. (**K–N & P–S**) Immunohistochemical analysis of Tbr2^+ve^ UBC progenitors in EGL and Tbr2^+ve^ glutamatergic progenitors in DCN in sagittal sections of E14.5 cerebellum. (**O**,**T**) Schematic showing the regions analyzed and represented in (**K**–**N & P**–**S**) and quantification of Tbr2^+ve^ UBC progenitors in EGL(**O**) and Tbr2^+ve^ cells in DCN (**T**). Data expressed as Mean ± SD, *n* = *3.* Scale Bar = 25 μm (**A**–**D**), (**F**–**I**), (**P**–**S**), 50 μm (**K**–**N**).

**Figure 6:**
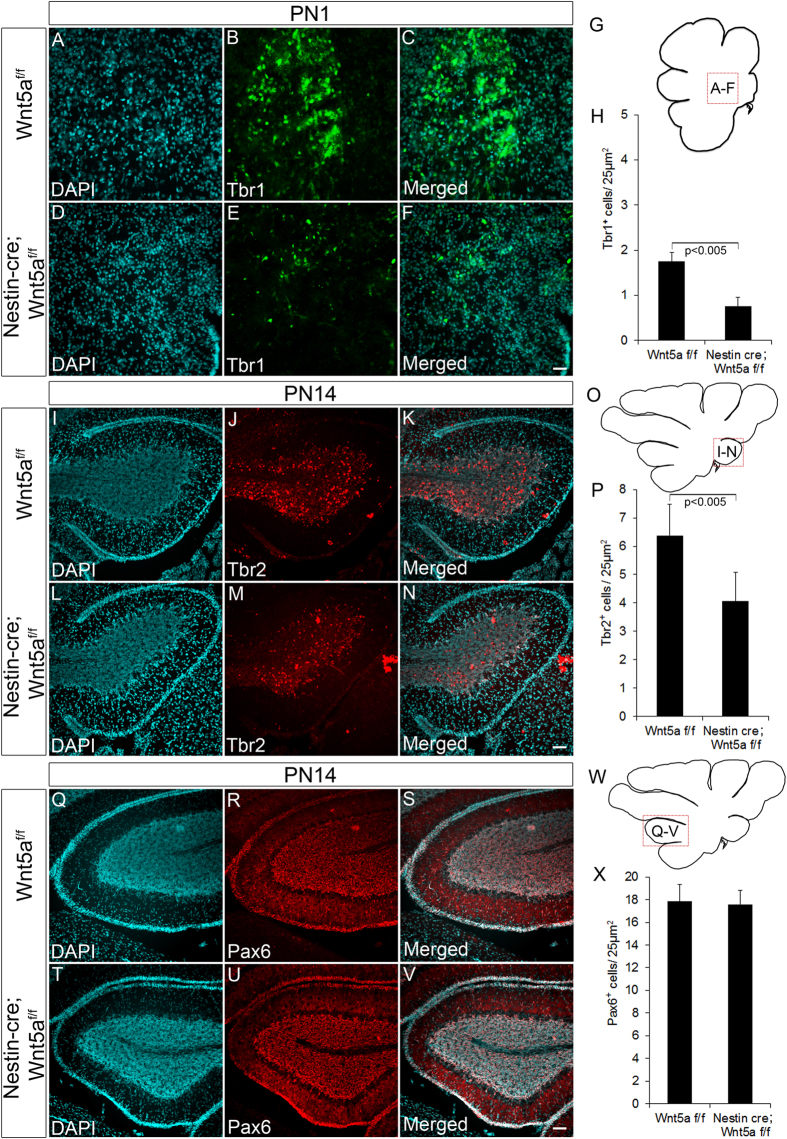
Wnt5a regulates the generation of glutamatergic neurons during early stages of development but not during the late postnatal stages. (**A**–**F** & **G**) Immunohistochemical analysis of Tbr1^+ve^ progenitors at PN1 stage. Schematic (**G**) indicates the region of cerebellum represented in (**A**–**F**). (**H**) Graph indicates quantification of Tbr1^+ve^ progenitors (**I**–**N** & **O**) Immunohistochemical analysis of Tbr2^+ve^ progenitors at PN14 stage. Schematic (**O**) indicates the region of cerebellum represented in (**I**–**N**). (**P**) Graph indicates quantification of Tbr2^+ve^ progenitors (**Q**–**V** & **W**) Immunohistochemical analysis of Pax6^+ve^ progenitors at PN14 stage. Schematic (**W**) indicates the region of cerebellum represented in (**Q**–**V**). (**X**) Graph indicates quantification of Pax6^+ve^ progenitors at PN14 stage. Data expressed as Mean ± SD, *n* = *3.* Scale Bar = 25 μm.

Our previous results have clearly shown the requirement of Wnt5a in proper development of GABAergic neurons in the cerebellum. These observations also prompted us to check if the development of glutamatergic neurons were also dependent on Wnt5a expression during embryonic stages. Glutamatergic neurons form the other major neuronal subtype in cerebellum and Math1^+ve^ progenitors in the RL sequentially gives rise to three different classes of glutamatergic neurons. Glutamatergic neurons of DCN being first are generated during early embryonic period (before E14.5)^40^, followed by UBCs and granule neurons during late embryonic (E14.5 to E19.5) and postnatal stages^41^,^42^. The DCN progenitors by E14.5 migrate from RL to populate the nuclear transitory zone (NTZ) where these progenitors express T-box transcription factors Tbr1 and Tbr2 during the early stage while in the later stages they are exclusively marked only by Tbr1 expression. Since Wnt5a cKOs displayed prominent reduction in the NTZ size, suggesting defective DCN neuron genesis. We next analyzed the expression of Tbr1 and Tbr2 at E14.5 by immunohistochemical analysis. A significant reduction (p < 0.005) in number of Tbr1^+ve^ cells was observed in Wnt5a cKOs as compared to the Wt counterparts indicating a severe reduction in DCN population (Fig. 5A–D & E). Further reduction in Tbr1^+ve^ progenitors was accompanied by marked reduction (p < 0.005) in number of Tbr2^+ve^ progenitors in NTZ thereby corroborating with the early observation (Fig. 5P–S & T). In addition to the reduction in Tbr2^+ve^ cells in NTZ, interestingly we observed a significant reduction in Tbr2 expressing cells in developing EGL and in cells migrating from RL towards white matter (Fig. 5K–N & O). Besides marking the DCN progenitors, Tbr2 at E14.5 also marks the UBC progenitors in the RL, developing EGL and those streaming from the RL towards white matter but serves as marker only for UBCs during late embryonic and postnatal stages. Thus, reduction in Tbr2^+ve^ cells in EGL of the mutants at E14.5 strongly points to the essential role of Wnt5a in UBC’s generation. Since the generation of two major glutamatergic subtypes were affected by loss of Wnt5a, we were curious to understand its role in regulating the genesis of granule neuron progenitors. Pax6, a paired box transcription factor marks all the granule neurons throughout its development (from progenitor to the differentiated state). Immunohistochemical analysis revealed a significant reduction in number of Pax6^+ve^ glutamatergic neuronal progenitors in the developing EGL at E14.5 (Fig. 5F–I & J) thereby, strongly indicating the requirement of Wnt5a signaling in the generation of granule neurons as well. As the generation and maturation of these neurons nears completion during postnatal stages we further analyzed the expression of these markers during late development. Consistent with the reduction in DCN progenitors at E14.5 we observed a significant reduction in Tbr1^+ve^ neurons in medial deep nuclei at PN1 thereby, pointing to the fact that loss of Wnt5a results in depletion of DCN population (p < 0.005, Fig. 6A–F & G,H). Similarly, in line with reduction in Tbr2^+ve^ UBC progenitors, we noted a significant reduction (p < 0.005) in Tbr2^+ve^ neurons at PN14 stage also (Fig. 6I–N & O,P), which additionally laid emphasis on role of Wnt5a in ensuring correct generation of UBC’s. But in striking contrast to these cell types, Pax6 expressing granule neurons did not show any significant reduction at PN14 as observed in early embryonic stages (Fig. 6Q–V & W,X). This result was in corroboration with our previous histological analysis where the EGL did not show a significant thinning as the other cortical layers at PN7 (Fig. 2T). Thus, these observations clearly indicate that Wnt5a is one of the important factors that is required for regulating the generation of UBC’s and glutamatergic neurons of the DCN but is dispensable for the genesis of granule neurons.

### Wnt5a mutants display defects in proliferation

**Figure 7:**
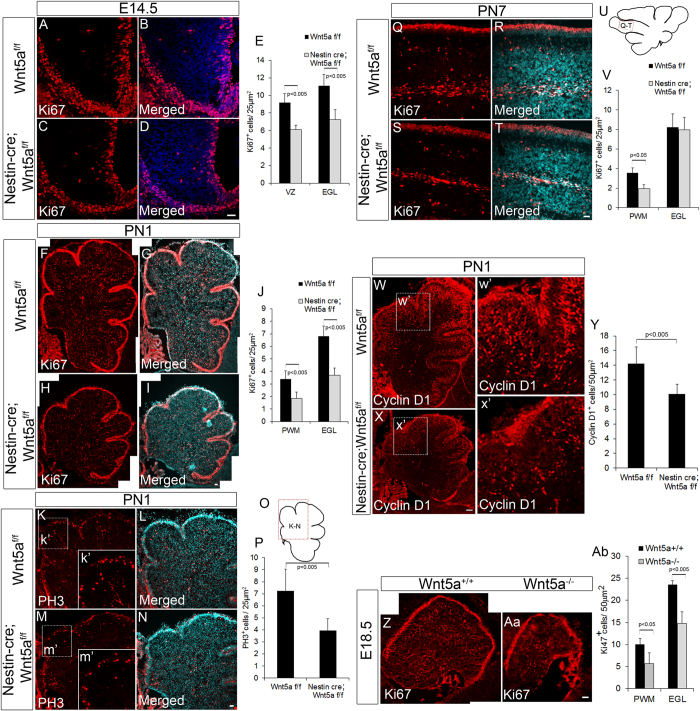
Wnt5a regulates proliferation of VZ and RL progenitors in discrete manner during embryonic and postnatal development. (**A**–**D**) Immunostaining with proliferation marker Ki67 at E14.5. (**E**) Graph shows the quantification of Ki67^+ve^ cells at E14.5. (**F**–**I**) Image shows Ki67 staining at PN1 stage in Wnt5a cKO and control. (**J**) Graph shows the quantification of Ki67^+ve^ cells at PN1. (**K**–**N**) Immunostaining with PH3 at PN1. (**O**) Schematic indicates the area represented in the image (**K**–**N**). (**P**) Graph indicates quantification of PH3^+ve^ cells. (**Q–T**) Image shows Ki67 staining at PN7 stage in Wnt5a cKO and control. (**U**) Schematic indicates the area represented in the image (**Q**–**T**). (**V**) Graph indicates quantification of Ki67^+ve^ cells. (**W–X**) Immunohistochemical analysis with Cyclin D1 in sagittal sections (PN1) of Wnt5a^f/f^ control and Nestin-*Cre;* wnt5a^f/f^ cKO (**w’ & x’**) represents specific magnified area of W and X. (**Y**) Graph indicates the quantification of Cyclin D1^+ve^ cells at PN1. (**Z–Aa**) Immunostaining with Ki67 in E18.5 Wnt5a^+/+^ control and Wnt5a^−/−^ null mutants. (**Ab**) Graph indicates quantification of Ki67^+ve^ cells both in PWM and EGL of Wnt5a^+/+^ and Wnt5a^−/−^ null mutants. Image (**F**–**I**) and (**W**–**X**) were generated by stitching together multiple images using Photoshop software. Data expressed as Mean ± SD, *n* = *3.* Scale Bar = 25 μm (**A**–**D**,**F**–**I**,**K–N** & **Q**–**T**), 50 μm (**W**–**X** & **Z**–**Aa**).

**Figure 8:**
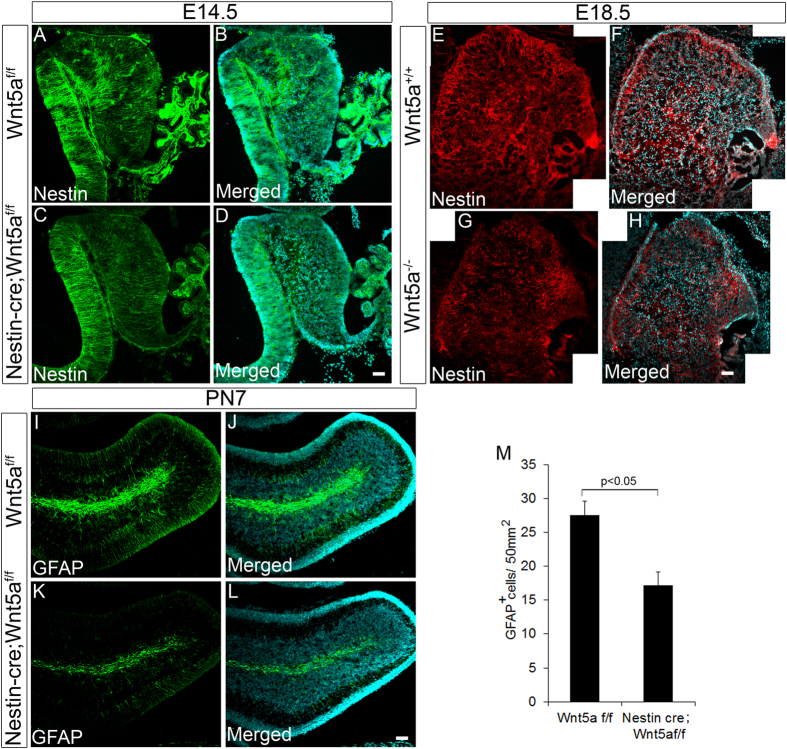
Loss of Wnt5a results in depletion of neural progenitor cells. (**A**–**D**) Immunostaining with neural progenitor marker Nestin at E14.5 stage in Wnt5a^f/f^ control and Nestin-*cre;* Wnt5a^f/f^ cKOs. (**E**–**H**) Immunostaining with neural progenitor marker Nestin at E18.5 stage in Wnt5a^+/+^ control and Wnt5a^−/−^ null mutants. (**I**–**L**) Immunostaining with neural progenitor marker GFAP at PN7 stage in Wnt5a^f/f^ control and Nestin-*cre;* Wnt5a^f/f^ cKOs. (**M**) Graph shows the quantification of GFAP^+ve^ cells at PN7. Data expressed as Mean ± SD, n = 3. Scale Bar = 25 μm.

Wnt5a signaling has been previously reported to promote survival, proliferation and differentiation of neural progenitors in different regions of the brain in a context dependent manner^23^,^25^,^26^. Since, we observed a significant reduction in size and number of GABAergic and glutamatergic neurons, we next sought to determine if the reduction was due to reduced proliferation or apoptosis. In order to address this question, we carried out TUNEL assay which did not reveal any observable difference in number of apoptotic cells between Wnt5a cKOs and Wt controls at any stage of development (E14.5, PN1 and PN7, Fig. S2). Therefore, we next assessed if loss of Wnt5a can result in defective proliferation of cerebellar progenitors. Immunohistochemical labeling of midsagittal sections at E14.5 stage with proliferation marker, Ki67 (marks the cells in G1, S and M phase) revealed significant reduction (p < 0.005) in number of proliferating cells both in VZ and EGL of the Wnt5a cKOs as compared to the Wt siblings (Fig. 7A–D & E). Since, we continued to observe a reduction in the size of the cerebellum at early postnatal stage (PN1), we analyzed the rate of proliferation in EGL and PWM. Our results showed a significant reduction (p < 0.005) in the number of Ki67^+ve^ proliferating cells at both germinal centers compared to the Wt counterparts (Fig. 7F–I & J). Consistently, we also observed a significant reduction (p < 0.005) in number of phospho histone 3 (PH3^+ve^) mitotic cells in the Wnt5a cKOs (Fig. 7K–N & O,P). Since Wnt5a cKOs displayed a significant reduction in number of proliferating progenitors, we next assessed the expression of cyclin D1 in conditional mutants as extracellular signaling molecules are well known to mediate proliferation through the regulation of D type cyclins^43^. We observed a significant reduction in number of Cyclin D1 expressing cells in mutants as compared to Wt control (Fig. 7W,w’,X,x’ & Y). Similarly, we also observed significant reduction in Ki67^+ve^ (Fig. 7Z,Aa & Ab) and PH3^+ve^ (data not shown) proliferating progenitors and also reduction in nestin expressing progenitor cells in E18.5 Wnt5a^−/−^ null mutants as compared to Wt control (Fig. 8E–H) which strongly supports the requirement of Wnt5a for maintaining normal number of proliferating progenitors.

On the other hand, at PN7 we did not observe thinning of Ki67^+ve^ outer EGL and quantitative analysis also did not reveal significant reduction in Ki67^+ve^ granule neuron progenitors, while there was a significant reduction in the number of Ki67^+ve^ cells in the PWM (Fig. 7Q–T & U,V) indicating the differential requirement of Wnt5a for mediating proliferation.

Further in order to assess if the defects caused are due to reduction in early progenitor population in response to loss of Wnt5a, we analyzed the expression of nestin, a radial glial progenitor marker that marks VZ progenitors of cerebellum using immunohistochemical analysis at E14.5. Intriguingly we observed reduction in density of the nestin fibers in conditional mutants at E14.5 (Fig. 8A–D) and in Wnt5a^−/−^ null mutants at E18.5 (Fig. 8E–H) as compared to their respective controls indicating reduction in radial glial progenitor population. Similarly, a significant reduction in Pax6^+ve^ RL progenitors in cKO’s at E14.5 (Fig. 5F–I & J) and in Wnt5a^−/−^ null mutants at E18.5 (Fig. S3A–C) also indicated the reduction in early progenitor pool in both the germinal zones upon loss of Wnt5a. Thus, these results clearly indicated that the reduction in progenitor pool is an early event. To further ascertain if there is reduction in proliferating radial glial progenitor population of the PWM, we carried out immunohistochemical analysis with GFAP at PN7 stage (Wnt5a cKO). GFAP marks the radial glial progenitor in PWM of the cerebellum during postnatal stages which subsequently gives rise to the Pax2^+ve^ GABAergic interneurons^44^. As observed during the early development we noted a significant decrease in the GFAP^+ve^ progenitor cells in the PWM (Fig. 8I–L & M). In corroboration with this observation, we have previously noted a marked reduction in Pax2^+ve^ cells in Wnt5a conditional mutants at PN7. Thus, these results convincingly indicate that loss of Wnt5a causes marked reduction in the progenitor pool in PWM which results in significant reduction in Pax2^+ve^ interneurons. Collectively, these results indicate that Wnt5a supports proliferation of VZ progenitors throughout development while, it is primarily required to support proliferation of EGL progenitors during early development.

### Wnt5a regulates the expression of proliferative genes in a β-catenin independent manner

**Figure 9:**
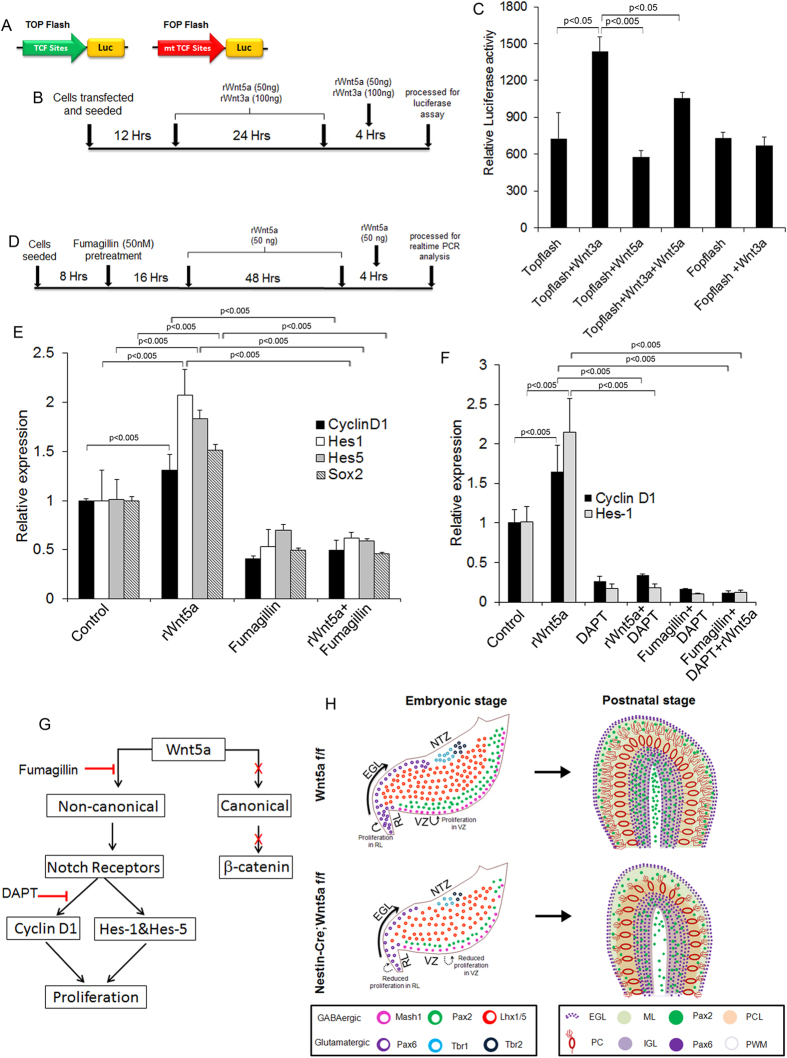
Wnt5a non-canonically regulates genes associated with cerebellar progenitor proliferation. (**A**) Schematic of TOPFlash and FOPFlash luciferase constructs. (**B**) Schematic represents the protocol followed for dual luciferase assay in primary cerebellar culture. (**C**) Dual luciferase assay with TOPFlash and FOPFlash luciferase reporter constructs. (**D**) Schematic shows the experimental procedure followed for gene expression analysis using real time PCR. (**E**) Real time PCR analysis of Cyclin D1, Sox2, Hes1 and Hes5 expression upon treatment with rWnt5a protein and non-canonical Wnt signaling blocker fumagillin. (**F**) Real time PCR analysis of cyclinD1 and Hes1 upon rWnt5a, DAPT and fumagillin treatment. (**G**) Schematic showing the pathway for activation of cyclin D1 by Wnt5a. (**H**) Schematic showing effect of Wnt5a loss during embryonic and postnatal stages on development of cerebellum. Data expressed as Mean ± SD, *n* = *3.*

Wnt5a is capable of mediating both canonical and non-canonical Wnt signaling based on the receptor context^45^, thus we next were curious to understand whether Wnt5a mediates the function in a canonical or non-canonical manner in this context. For this we transfected primary cerebellar cells with TOPFlash or FOPFlash (Fig. 9A) construct and exposed them to rWnt5a or rWnt3a for 24 hours and carried out luciferase assay (Fig. 9B). Treatment of primary cerebellar cells with rWnt5a upon transfection of TOPFlash luciferase reporter construct did not show any discernible change in the luciferase activity (Fig. 9C). Whereas, treatment with known canonical Wnt ligand Wnt3a resulted in significant up-regulation (p < 0.05) of luciferase activity compared to TOPFlash controls thus, strongly pointing towards non-canonical mode of action of Wnt5a (Fig. 9C). In agreement with our previous *in vivo* results we also observed an increase in expression of Cyclin D1, a gene associated with proliferation and Sox2, a neural stem cell marker in primary cerebellar culture upon rWnt5a protein exposure (Fig. 9D & E) indicating that Wnt5a promotes proliferation while blocking non-canonical Wnt signaling using fumagillin resulted in decreased Cyclin D1 expression (Fig. 9E). Further treatment with rWnt5a and fumagillin combination also showed a significant down regulation (p < 0.005 respectively) of Sox2 and Cyclin D1 compared to the cells exposed to rWnt5a alone (Fig. 9E). We next assessed the expression of Notch target genes Hes1 and Hes5 as they have been implicated to play essential role in regulating proliferation^46^. Exogenous addition of rWnt5a protein to primary cerebellar culture cells resulted in up-regulation of Hes1 and Hes5 while blocking non-canonical Wnt signaling with fumagillin lead to marked down-regulation of these genes (Fig. 9E). Taken together, our results confirmed the regulation of proliferation related genes by non-canonical Wnt5a signaling.

Previously Wnt5a has been shown to regulate Hes1 and Hes5 transcriptional activity through Notch receptor gene expression regulation in a β-catenin independent manner^47^. Thus, in order to understand if Wnt5a mediates the regulation of proliferation in a Notch dependent or independent manner we analyzed the expression of Hes1 and Cyclin D1 upon blocking Notch signaling with γ-secretase inhibitor DAPT. DAPT exposure resulted in down-regulation of cyclinD1 and Hes1 (Fig. 9F) thereby, pointing to the role of Notch in regulation of these genes as previously reported by several other studies^48^,^49^. But interestingly, addition of exogenous rWnt5a protein resulted in up-regulated expression of these genes but it was unable to exert a similar function in the presence of Notch inhibitor DAPT and expression of these genes remained markedly down-regulated (Fig. 9F). Since, we have shown the non-canonical activation of notch signaling through Wnt5a, we also wanted to understand whether the classical non-canonical downstream targets of Wnt5a such as JNK and CamkII are activated by Wnt5a during cerebellar development. For this we treated CGN cultures with rWnt5a and JNK inhibitor SP600125 and carried out real-time expression analysis of Cyclin D1. Our results showed that exposure of CGN cultures to JNK blocker SP600125 did not bring about any significant decrease in expression of Cyclin D1, ruling out the involvement of JNK in this context (Fig. S4). Although we could rule out the connection between Wnt5a and JNK, there is always a possibility that Wnt5a can activate CamkII and there by activate Notch signaling^47^. Together, our data indicates that Wnt5a regulates the expression of proliferative genes in a Notch dependent manner.

## Discussion

Several studies have demonstrated the pleiotropic role of Wnt5a signaling in mediating neural progenitor proliferation and differentiation in various regions of the brain in a context dependent manner^25^,^26^. However, a comprehensive description of its expression pattern and functional relevance in cerebellar development has remained unclear. Therefore, in the present study for the first time we demonstrate the novel role of non-canonical Wnt5a signaling in regulating neurogenesis by mediating proliferation of cerebellar progenitors in a dynamic manner. At molecular level we provide evidence for Notch dependent activity of Wnt5a in regulating the expression of genes associated with proliferation (Fig. 9G). Thus, our study proves Wnt5a to be one of the key signaling molecules that ensures normal cerebellar growth and development.

In cerebellum, different types of GABAergic and glutamatergic neurons are generated during embryonic and postnatal development^50^,^51^. Glutamatergic and GABAergic neurons of the DCN, Purkinje neurons and some of the GABAergic interneurons are generated during embryonic period while majority of the GABAergic interneurons and granule neurons are generated during the postnatal development^41^,^42^,^52^. Our results indicate a significant reduction in number of Ki67^+ve^ proliferating progenitors in both the primary and secondary germinal zones of the cerebellum at E14.5 and PN1 stages. Strikingly, unlike E14.5 and PN1, at PN7 stage we observed a significant reduction in number of proliferating progenitors in PWM but not in EGL. Consequently, impaired proliferation resulted in severe reduction of Tbr1^+ve^ glutamatergic neurons of the DCN, Tbr2^+ve^ UBC’s, Lhx1/5^+ve^ Purkinje cells and Pax2^+ve^ GABAergic interneurons both during early and late postnatal development. Conversely, Pax6^+ve^ CGNPs were depleted in early stage but not during late postnatal stage. In accordance with the finding that proliferation of EGL progenitors at PN7 stage was not significantly altered by loss of Wnt5a since, EGL in Wnt5a cKOs did not display significant reduction in thickness. Additionally, the fact that the reduction in size was not prominently pronounced as development progressed also strongly indicated that the proliferation of granule neuron progenitors at later stages is not impaired in wnt5a conditional mutants. Since, granule neuron progenitor’s are well known to compensate for the mitotic insult, we cannot totally rule out the possibility of Wnt5a’s mitogenic effect on granule neuron progenitors^53^,^54^,^55^.

Further, to date various studies have implicated the role of Wnt β-catenin signaling, Notch and Shh in mediating proliferation of cerebellar progenitors at different stages^19^,^56^,^57^,^58^,^59^. Wnt β-catenin signaling has been shown to promote the proliferation and inhibit the differentiation of the VZ progenitors during early developmental stage while it promotes only the differentiation of EGL progenitors instead of proliferation thereby, playing a crucial role in cerebellar development^19^. Notch signaling components have been shown to be expressed in germinal centers during embryonic and postnatal development and have been implicated in regulating proliferation of cerebellar progenitors^58^,^60^. Another major signaling pathway that prominently regulates proliferation in cerebellum is Shh^59^,^61^ that promotes the proliferation of VZ progenitors both during embryonic and postnatal development. In addition, it is also known to regulate the proliferation of EGL progenitors right from E16.5 stage. Even though the role of Shh in regulating proliferation of EGL progenitors in later stages of development is quite clear, the factors involved in the regulation of proliferation during the early stages still remains an important question. Thus, our finding is of prime significance as our results confirm the role of Wnt5a in mediating proliferation of RL progenitors during the early development of cerebellum.

Several line of evidences point to co-operative function of different signaling pathways in regulating proliferation and differentiation of cerebellar progenitors during development and disease condition^19^,^62^. Transcriptional regulation of Cyclin D1 by β-catenin TCF signaling is well evidenced and it could be quite possible to speculate Wnt5a to mediate β-catenin dependent canonical pathway as it is capable of activating both β-catenin independent and dependent pathways in a context dependent manner^45^. However, Wnt5a was unable to activate TCF promoter activity thereby clearly ruling out this speculation. Although our *in vitro* data excludes activation of canonical downstream targets of Wnt5a, we cannot completely rule out activation of β-catenin *in vivo* during cerebellar development. Further in order to gain molecular insight into the mechanism through which Wnt5a mediated proliferation, we analyzed the role of Notch signaling since, Wnt5a has been shown to activate notch signaling through CamkII^47^. Our data clearly demonstrated Notch dependent activation by Wnt5a, but the regulation of Notch signaling by Wnt5a might be a direct or indirect effect and further investigation would be required in order to understand how these two pathways interact.

Besides reduction in rate of proliferation during conditional ablation of Wnt5a lead to improper dendritic branching and stunting of Purkinje neurons. Evidences from previous studies prove the essential role of granule neurons in mediating Purkinje cell dendritic arborisation^34^. Therefore, loss or reduction in factors secreted by granule neurons may be a cause for these defects. Additionally, defects in granule neuron progenitor proliferation in early stages have also known to result in stunting and poor dendritic arborisation of Purkinje cells in cerebellum^63^. Taking into account these observations the defects in granule neuron progenitor proliferation might be a major cause for defects in Purkinje cell maturation in Wnt5a conditional mutants.

We would also like to relate our findings to medulloblastoma since, deregulation of Wnt signaling pathway has been one of the major cause for medulloblastoma^64^. Though only expression of Wnt5a in medulloblastoma tumor samples has been reported recently, its role in medulloblastoma prognosis remains unclear^65^. Previously, role for non-canonical Wnt signaling in type-c and d medulloblastoma tumors has been indicated. It has also been suggested that cells of VZ origin might be affected^66^. As our results confirms the role of Wnt5a in regulating proliferation of VZ progenitors and RL progenitors during early development it is possible that Wnt5a signaling might be one of the important signaling that could drive medulloblastoma tumorigenesis. Thus, better understanding of this signaling pathway would prove to be extremely useful. Putting together our findings, (Fig. 9H) we conclude that Wnt5a expression is critical for proliferation of RL and VZ progenitors and Purkinje cell dendritogenesis during early embryonic development resulting in retarded development of cerebellum during postnatal stages.

## Methods

### Generation of Nestin-*cre* Wnt5a conditional knockout and Wnt5a^−/−^ null mice

Wnt5a conditional knockout (cKO) embryos and postnatal pups were generated by crossing Nestin-*Cre* mice with Wnt5a^f/f^ mice and kindly provided by Dr. Rejji Kuruvilla (Johns Hopkins University, USA). The protocol for generation of Nestin-*Cre*; Wnt5a^f/f^ cKOs and procedures relating to animal care were approved and conformed to Johns Hopkins University Animal Care and Use Committee (ACUC) and NIH guidelines. The embryos and postnatal pups were genotyped and checked for deletion of exon2 of *Wnt5a* gene. Loss of Wnt5a expression in Nestin-*Cre*; Wnt5a^f/f^ cKOs was also confirmed by *In situ* hybridization. Wild-type Wnt5a^f/f^ mice were used as controls. E18.5 Wnt5a^−/−^ null embryos were generated and provided by Dr. Kerstin Buttler (University Medicine Göttingen, Germany). E18.5 Wnt5a^+/+^ embryos were used as controls. The protocol for generation of E18.5 Wnt5a^−/−^ KO embryos were approved and conformed to the animal care and ethical guidelines of University Medicine Göttingen, Germany.

### *In situ* hybridization analysis

*In situ* hybridization analysis was carried out using specific DIG labeled antisense and sense probes generated from pIRES-hrGFP-1a-Wnt5a vector (Kind gift from Dr.Yingzi Yang, NIH, Bethasda, MD). Brain tissue was dissected out from mouse embryos and postnatal pups and fixed with freshly prepared 4% PFA for overnight at 4 °C. This was followed by dehydration with 30% sucrose, samples were embedded in O.C.T (TissueTek) and 20 μm thick serial sections were taken on superfrost positively charged slides (Electron Microscopy Sciences). Tissue sections were again fixed with 4% PFA for 10 min and acetylated using 0.25% acetic anhydride in 0.1 M Triethanolamine. Following pretreatment, sections were hybridized with DIG labeled RNA probe at 1.5 μg/ml concentration for overnight in a humidified chamber at 68 °C. Sections were then washed with 0.2X SSC buffer at 65 °C, blocked with 10% normal goat serum in TBS for 1 hr at RT and then incubated with Anti DIG antibody conjugated to alkaline phosphatase (1:5000) for overnight at 4 °C. On day three, sections were washed and incubated with NBT/BCIP (SIGMA) solution till the color developed. Slides were rinsed briefly in 1X PBS, fixed using formaldehyde solution and mounted using Fluoromount-G (Electron Microscopy Sciences). *In situ* hybridization protocol was approved and carried out according to the guidelines of institutional animal ethics committee (IAEC) of Rajiv Gandhi Center for Biotechnology.

### Real time RT-PCR analysis

For temporal analysis of Wnt5a expression, total RNA was isolated from whole cerebellar tissue samples using TRIZOL reagent and equal amount of RNA were converted to cDNA using superscript RT-II (Invitrogen). Real time PCR analysis was done using SYBR green mix (Biorad) and the values were normalized to the β-actin values^67^.

In case of primary cerebellar culture, total RNA was extracted from the primary cells using Qiagen RNeasy Mini kit according to manufacturer’s protocol and 1 μg RNA was converted to cDNA followed by real time PCR analysis using specific primers for different genes of interest (Table S1).

### Immunohistochemistry

For immunohistochemical analysis brain samples were dissected, fixed in 4%PFA for overnight at 4 °C and dehydrated using 30% sucrose^68^. Samples were then embedded in O.C.T (TissueTek) and 16 μm thick sections were taken on gelatin-coated slides. Sections were washed in 1XPBS and blocked with 5% normal goat serum (Sigma-Aldrich) containing 0.2% or 0.4% TritonX-100 (cytoplasmic or nuclear antigens respectively) for 1 hr at room temperature. Thereafter, primary antibodies were diluted in the respective blocking solution and incubated for overnight at 4 °C followed by incubation with appropriate secondary antibody for 90 minutes at room temperature. Finally, slides were counterstained with DAPI and mounted using Fluoromount G. Antigen retrieval using 10 mM sodium citrate buffer, pH-6 for 10 min was performed for all the antibodies except Calbindin and Parvalbumin. Primary antibodies used for the study were mouse anti-Ki67 (1:50, BD Biosciences-550609), mouse anti-PH3 (1:100, Abcam-mAB14955), rabbit anti-Cyclin D1 (1:50, Cell Signalling-2978), mouse anti-Lhx1/5 (1:50, DSHB- 4F2), mouse anti-Mash1 (1:50, BD Biosciences-556604), rabbit anti-Pax2 (1:50, Zymed laboratories-71-6000), rabbit anti-Pax6 (1:200, Millipore-AB2237), rabbit anti-Tbr2 (1:200, Abcam-ab23345), rabbit anti-Tbr1 (1:500, Abcam-ab31940), mouse anti-calbindin (1:1500, Sigma-C9848), rabbit anti-parvalbumin (1:500, Abcam-AB11427), mouse anti-VGlut1 (1:100, Chemicon-MAB5502) and mouse anti-VGlut2 (1:100, Chemicon-MAB5504), rabbit anti-Pax6 (1:200, Millipore-AB2237), mouse anti-GFAP (1:200, Sigma-G3893), rabbit antiTbr2 (1:200, Abcam-ab2345) and mouse anti-nestin (1:40, DSHB-Rat401). Secondary antibodies used were Sheep anti-mouse Cy3 (1:400, Jackson Immuno-Research), Goat anti-rabbit Cy3 (1:400, Jackson Immuno-Research), Goat anti-rabbit Alexa Fluor488 (1:200, Molecular Probes-A11008), Goat anti-mouse Alexa Fluor488 (1:200, Molecular Probes- A11001). After processing, sections were analyzed using upright fluorescent microscope (Olympus BX 61) and images were obtained using cooled CCD camera (Andor 885). Some of the images were analyzed using confocal microscope.

### Primary cerebellar culture

All animal experiments relating to primary culture were approved and carried out according to the guidelines of institutional animal ethics committee (IAEC) of Rajiv Gandhi Center for Biotechnology. Mouse cerebellar primary culture was done as described earlier^69^. Briefly, 3-day old mouse pups (PN3) were sacrificed by decapitation and the cerebellum was dissected out under sterile conditions. Tissue was chopped and treated with 0.05% Trysin- EDTA and DNase for 10 min at 37 °C in order to dissociate into single cells. After dissociation, single cells were resuspended in Neurobasal medium containing B27, 1× glutamax, 430 μM glucose, 10 mM KCl and 10% FBS and plated at a density of 1.5 × 10^6^ cells per well of 24 well plate coated with 10 μg/ml of poly-D-lysine (Sigma Aldrich) and 5 μg/ml laminin (BD Biosciences). The cells were maintained in serum containing media for overnight, then changed to serum free media and the cultures were maintained for 4 days. Treatment with 50 ng rWnt5a protein (R&D) was initiated on day 2 and cells were fed with fresh media containing rWnt5a protein every 24 hrs. Further, 4 hrs priming was given with rWnt5a protein prior to collection of cells. Pretreatment for 16 hrs with Fumagillin (50 nM) was given before rWnt5a protein treatment commencement and then cells were cultured with or without Wnt5a.

### Dual-luciferase assay

Dual Luciferase assay was done according to manufacturer’s protocol (Promega, Cat. no. E1910) using Luminometer (TD20/20, Promega). Briefly, primary cerebellar cells were electroporated with TOPFlash (Plasmid #12457, Addgene), β-catenin reporter with 8X TCF/LEF binding sites and FOPFlash (Plasmid #12456, Addgene), β-catenin reporter with 8X mutated TCF/LEF binding sites vector along with Renilla luciferase and were seeded in a 24 well plate^67^. After 12 hrs of electroporation, cells were treated with 50 ng and 100 ng of rWnt5a and rWnt3a protein respectively for 36 hours. Then cells were lysed using 1X passive lysis buffer for 30 min followed by centrifugation at 5000 g for 5 min and the assay was carried out using supernatant. Three replicates were used for each sample and values of Renilla luciferase were used to normalize the discrepancies in transfection efficiency between samples.

### TUNEL assay

Cell death was analyzed using fluorescien *in situ* TUNEL labelling kit (Roche) according to manufacturer’s protocol with mild modification. 20 μm cryosections were boiled for 10 min in 10 mM sodium citrate buffer, pH6 following which slides were rinsed in 1X PBS and incubated in TUNEL labeling mix containing the enzyme in a manufacturer recommended concentration for 1 hr at 37 °C in a humidified chamber. Slides were then washed in 1XPBS and counterstained with DAPI. Total number of TUNEL^+ve^ cells per cerebellar section was counted and average from 3 sections was plotted.

### Area measurement and cell counts

Area of sagittal cerebellar section was calculated for each developmental stage (E14.5, PN1 and PN7) after DAPI staining using Image J software from NIH (version 1.48). All quantifications were done blinded to the genotype. Each section was divided into equal bins (of same area) and cell counting was done using the Photoshop counting tool after immunohistochemical analysis with specific markers. For each stage littermates were analysed and all quantification was carried out in at least 3 sections per individual in minimum of 3 animals for each stage and the data was averaged.

### Statistical analysis

Statistical significance between the groups was calculated by unpaired student’s *t*-test and p < 0.05 was considered statistically significant. Statistical significance of multiple groups was analyzed using Tukey HSD Post-hoc test. All results are presented as mean ± SD.

## Additional Information

**How to cite this article:** Subashini, C. *et al*. Wnt5a is a crucial regulator of neurogenesis during cerebellum development. *Sci. Rep.* **7**, 42523; doi: 10.1038/srep42523 (2017).

**Publisher's note:** Springer Nature remains neutral with regard to jurisdictional claims in published maps and institutional affiliations.

## Supplementary Material

Supplementary Information
