# Supplementary material for: Wnt5a is a crucial regulator of neurogenesis during cerebellum development
Source: Sci Rep. 2017 Feb 16;7:42523. doi: 10.1038/srep42523 (PMC5311982; doi:10.1038/srep42523)
Supplement: Supplementary Information [file srep42523-s1.pdf]

Supplementary Information

Title: Wnt5a is a crucial regulator of neurogenesis during cerebellum development

Chandramohan Subashini[1], Sivadasan Bindu Dhanesh[1], Chih-Ming Chen[2], Paul Ann Riya[1], Vadakkath Meera[1], Thulasi Sheela Divya[1], Rejji Kuruvilla[2], Kerstin Buttler[3] & Jackson James[1*]

[1] *Neuro Stem Cell Biology Laboratory, Neurobiology Division, Rajiv Gandhi Centre for Biotechnology, Thiruvananthapuram, Kerala-695 014, India*

[2] *Department of Biology, Johns Hopkins University, 3400 N. Charles St., 224 Mudd Hall, Baltimore, MD 21218, USA*

[3] *Department of Anatomy and Cell Biology, University Medicine Göttingen, 37075-Göttingen, Germany*

Fig. S1

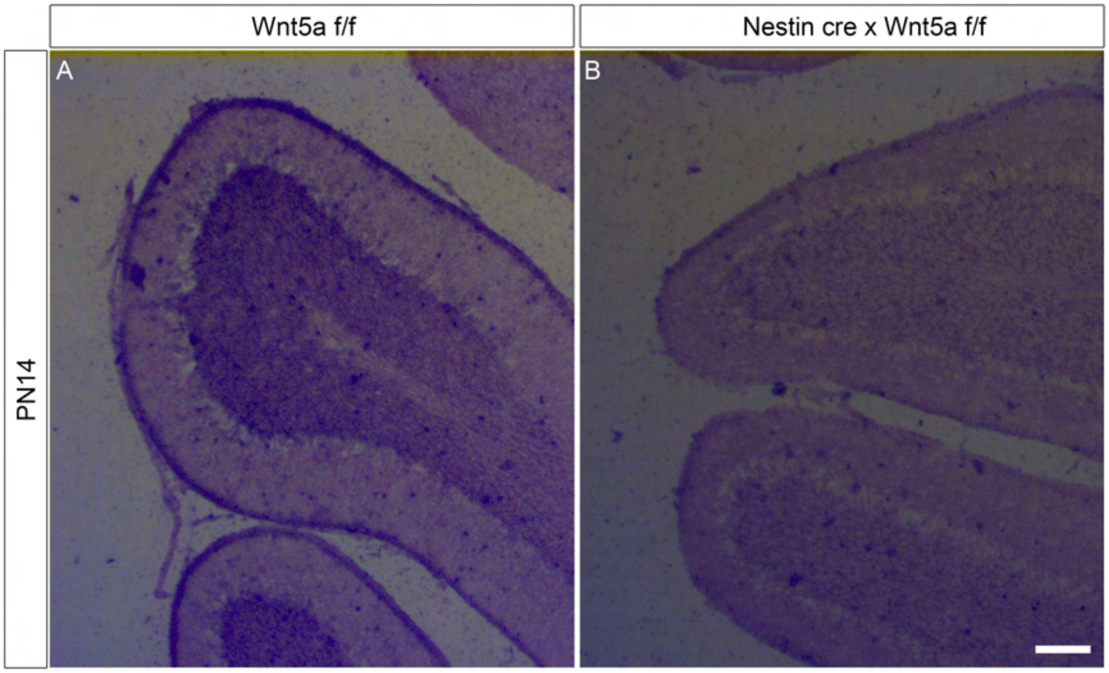

Fig. S2

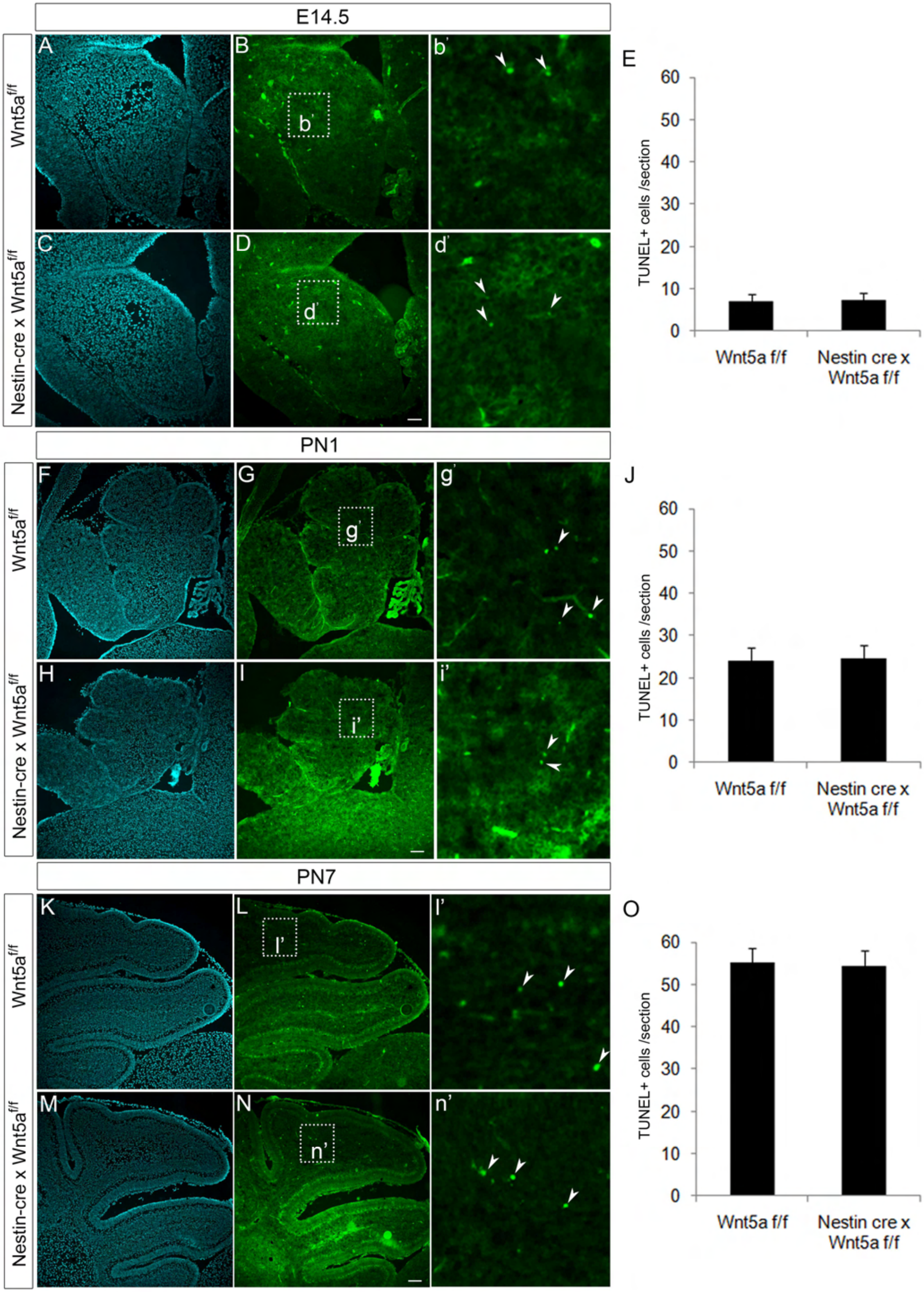

E14.5
A
B
b'
b'
C
D
d'
d'
Wnt5a f/f
Nestin-cre x Wnt5a f/f
E
TUNEL+ cells /section
60
50
40
30
20
10
0
Wnt5a f/f
Nestin cre x Wnt5a f/f
PN1
F
G
g'
g'
H
I
i'
i'
J
PN7
K
L
l'
l'
M
N
n'
n'
O

Fig.S3

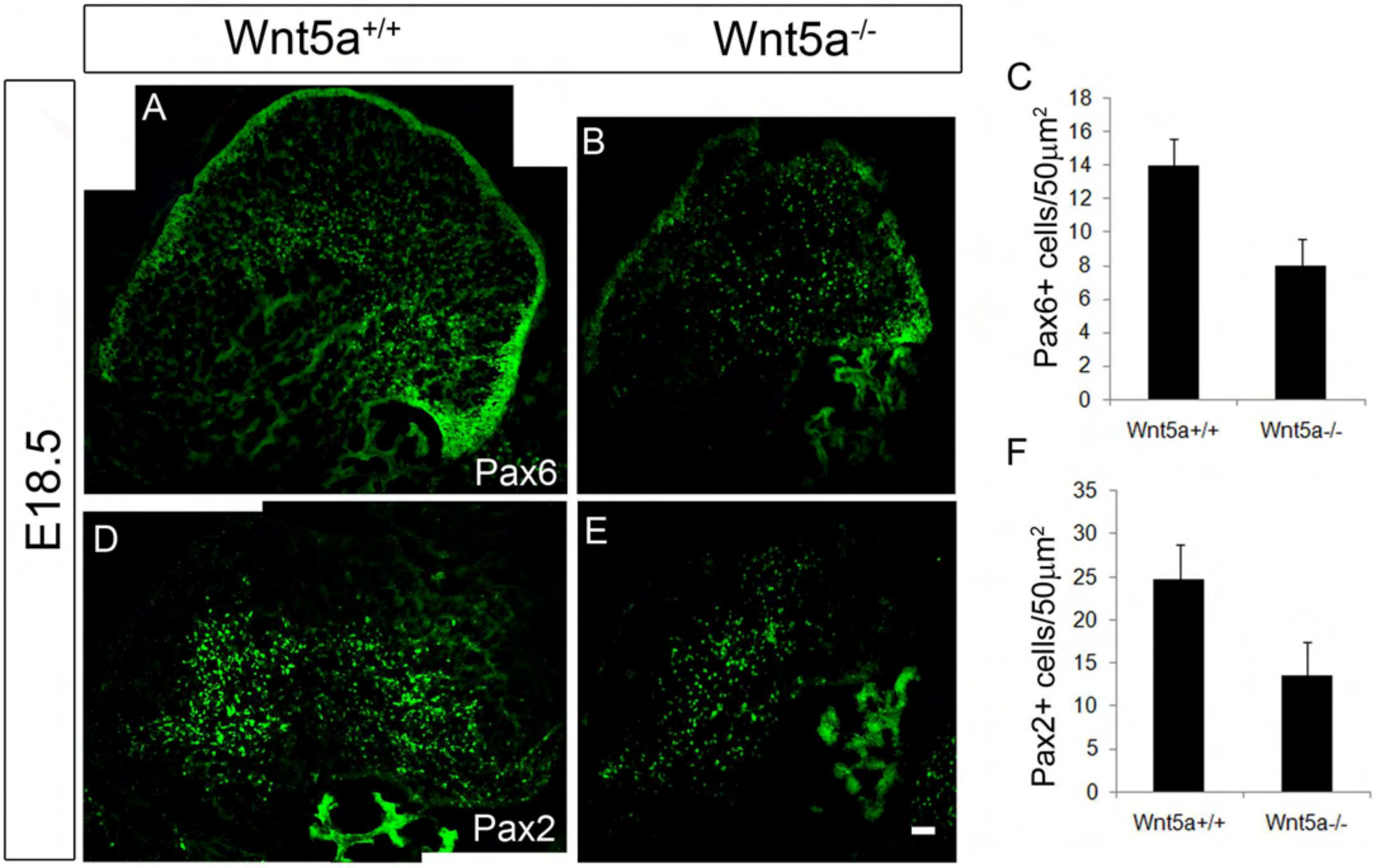
Wnt5a+/+
Wnt5a-/-
E18.5
A
B
Pax6
D
E
Pax2
C
Pax6+ cells/50μm2
0
2
4
6
8
10
12
14
16
18
Wnt5a+/+
Wnt5a-/-
F
Pax2+ cells/50μm2
0
5
10
15
20
25
30
35
Wnt5a+/+
Wnt5a-/-

Fig.S4

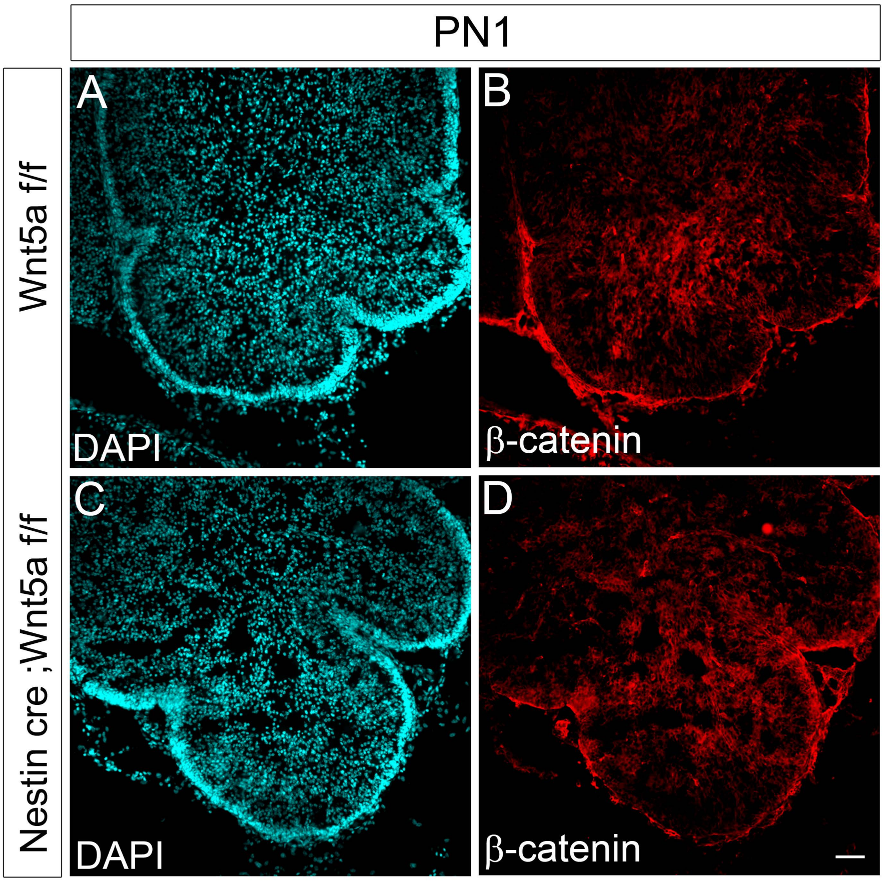
PN1
Wnt5a f/f
Nestin cre ;Wnt5a f/f
A
DAPI
B
β-catenin
C
DAPI
D
β-catenin

Fig.S5

A

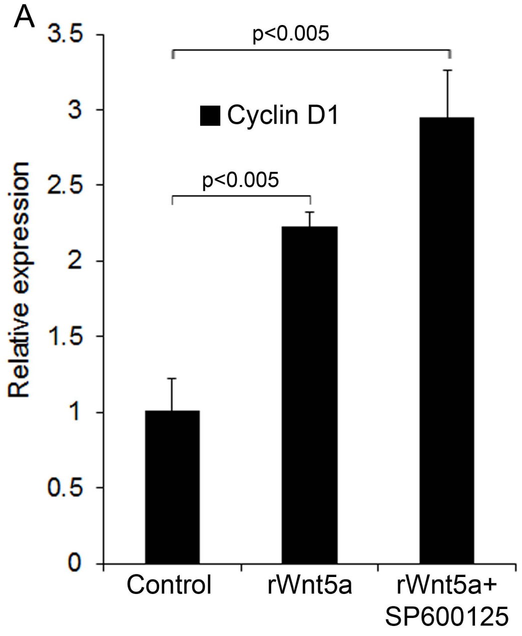

p<0.005
Cyclin D1
p<0.005
Relative expression
3.5
3
2.5
2
1.5
1
0.5
0
Control
rWnt5a
rWnt5a+ SP600125

Table S1: List of primers used for Quantitative RT-PCR analysis

Gene Name	Primer Sequence	Ann. Temp (°C)	Product Size (bp)
**β-actin**	F- AGACTTCGAGCAGGAGATG<br>R- CTTGATCTTCATGGTGCTAGG	56	322
**Hes-1**	F- TCAACACGACACCGGACAAAC<br>R- TTCATGCACTCGCTGAAGCC	56	295
**Hes-5**	F- CAAGGAGAAAAACCGACTGCG<br>R- GGTAAAGCAGCTTCATCTGCG	56	314
**Sox2**	F- CCAAGACGCTCATGAAGAAG<br>R- TGGTCATGGAGTTGTACTGC	60	288
**Cyclin D1**	F- GGAACTGCTTCTTCTGGTGAACAAG<br>R- TGGAGGGTGGGTTGGAATG	58	185

Supplementary Figure Legend

Figure S1: Wnt5a expression is lost in Nestin-*Cre* conditional knockout mutant cerebella. **(A-B)** *In situ* hybridization analysis using Wnt5a specific DIG labelled probes indicates loss of Wnt5a expression in Wnt5a cKO (B) as compared to Wt-type controls (A) at PN14 stage. Scale Bar = 50 µm.

Figure S2: Analysis of cell death in control and Wnt5a cKO using TUNEL assay. (A-E, F-J&K-O) TUNEL assay revealed no significant difference in number of $TUNEL^{+ve}$ cells at E14.5, PN1 and PN7 respectively. **(b'&d')** Magnified view of selected area in B and C. **(g'&i')** Magnified view of selected area in G and I. **(l'&n')** Magnified view of selected area in L and N. Arrowheads indicate the $TUNEL^{+ve}$ cells at each stage. Data expressed as Mean ± SD, *n=3*. Scale. Bar = 50 µm.

Figure S3: $Wnt5a_{-/-}$ null mutants exhibit severe reduction in GABAergic interneuron and granule neuron progenitors. (A-B) Immunohistochemical analysis using Pax6 antibody showed significant reduction in granule neuron progenitors in $Wnt5a^{-/-}$ null mutants at E18.5 compared to $Wnt5a^{+/+}$ controls **(C)** Quantitation of $Pax6^{+ive}$ cells in $Wnt5a^{+/+}$ control and $Wnt5a^{-/-}$ mutants. **(D-E)** Immunostaining of Pax2 at E18.5 stage in $Wnt5a_{+/+}$ and$Wnt5a_{-/-}$ sagittal cerebellar sections. **(F)** Quantitative analysis indicates significant reduction of $Pax2^{+ive}$ cells in $Wnt5a^{-/-}$ mutants as compared to $Wnt5a^{+/+}$ control. Data expressed as Mean ± SD, *n=3*. Scale. Bar = 50 µm.

Figure S4: Wnt5a cKo animals do not exhibit any difference in β-catenin expression. (A-D) Immunohistochemical analysis using β-catenin antibody did not show any significant difference in expression of β-catenin between PN1 $wnt5a^{f/f}$ and Nestin-*cre*;$Wnt5a^{f/f}$. Scale. Bar = 50 µm.

Figure S5: Wnt5a does not activate non-canonical JNK signalling during cerebellar development. (A) Treatment of CGN cultures with rWnt5a and JNK inhibitor SP600125 did not show any down regulation in expression of Cyclin D1 excluding the involvement of non-canonical Wnt5a mediated JNK activation. Data expressed as Mean ± SD, *n=3*.

Table S1: List of primers used
